# Interactions of Mycoplasma genitalium (Mg) and human papillomavirus (HPV) infections among couples

**DOI:** 10.1186/s12879-025-12334-y

**Published:** 2025-12-23

**Authors:** Nea Koskela, Julia Butt, Birgitta E. Michels, Kari Syrjänen, Seija Grenman, Tim Waterboer, Stina Syrjänen, Karolina Louvanto

**Affiliations:** 1 Department of Obstetrics and Gynecology, Faculty of Medicine and Health Technology, Tampere University, Arvo Ylpön katu, 34, Tampere, 33520 Finland; 2 German Cancer Research Center (DKFZ), Heidelberg, Germany; 3 SMW Consultants, Ltd, Kaarina, Finland; 4 Department of Obstetrics and Gynecology, Turku University Hospital, University of Turku, Turku, Finland; 5 Department of Oral Pathology, Institute of Dentistry, Faculty of Medicine, University of Turku, Turku, Finland; 6 Department of Pathology, Turku University Hospital, Turku, Finland; 7 Department of Obstetrics and Gynecology, Tampere University Hospital, Tampere, Finland

**Keywords:** *Mycoplasma genitalium*, HPV, PID, Antibody, Women, Men, Serology, Seropersistence, Oral, Genital

## Abstract

**Background:**

*Mycoplasma genitalium* is a sexually transmitted pathogen infecting the uterine cervix and causing pelvic inflammatory disease in women. Persistent high-risk human papillomavirus (HR-HPV) infections are important etiological agents in cervical and oral carcinogenesis. The potential interactions between *M. genitalium-* and HPV infections are incompletely studied.

**Methods:**

This study included 329 women and 135 of their male partners in the prospective Finnish Family HPV study, followed up for three years. Genital and oral scrapings and blood samples were collected at baseline and 12-, 24-, and 36-month follow-up visits. HPV-L1 IgG-antibodies to HPV6/11/16/18/45 and *M. genitalium* IgG-antibodies to MgPa N-term and rMgPa were assayed by multiplex serology, and HPV genotyping was performed by Multiplex Genotyping. Statistical analyses were conducted using the χ2-test, likelihood ratio, or Fisher’s exact test for categorical variables, and nonparametric tests for continuous variables. Crude and adjusted odds ratios (ORs) with 95% confidence intervals (95% CI) were calculated using the logistic regression models.

**Results:**

Persistent oral or genital HPV infections did not show any associations with the *M. genitalium* antibody levels. Incident oral HPV infections were significantly increased among women with high-levels of MgPa N-term antibodies, OR 4.14 (95%CI 1.10–15.52). *M. genitalium* antibodies were associated with an increased likelihood of seropositivity to HR-HPV during the follow-up, OR range from 2.66 to 4.62 (with 95%CI range of 1.01–11.00).

**Conclusion:**

*M. genitalium* serology seems to be unrelated to outcomes of genital HPV infections but might increase the incidence of oral HPV infections and the likelihood of HPV seropositivity among women.

**Supplementary Information:**

The online version contains supplementary material available at 10.1186/s12879-025-12334-y.

## Background

*Mycoplasma genitalium* (*Mg*) is a sexually transmitted pathogen, causing acute and chronic non-gonococcal urethritis (NGU) in males, infections in the uterine cervix, and pelvic inflammatory disease (PID) in women [1–3]. Co-infection with human papillomavirus (HPV) may enhance genital inflammation and contribute to HPV persistence or disease progression, underscoring the importance of studying these co-infections. According to a comprehensive recent meta-analysis, the prevalence of *Mg* was 1.3% and 3.9% in developed and developing countries, respectively [4]. This disparity may reflect differences in sexual health awareness, and higher rates of unprotected sexual activity, all of which facilitate sustained transmission. Apart from the well-defined clinical infections, *Mg* frequently causes asymptomatic infections, with reported rates varying widely across studies, which present challenges in diagnosis and treatment [1–4].

HPV infections represent the most frequent sexually transmitted infections (STIs) among women and men worldwide [5]. Different HPV genotypes are classified into high-risk (HR) and low-risk (LR) categories based on their oncogenic potential [6]. As the primary cause of cervical cancer [5, 7, 8], HPV is also implicated with several other genital and non-genital cancers in both genders, including multiple other anogenital and head and neck cancers [9]. Additionally, HPV infections often occur concomitantly with other STIs, including chlamydia, gonorrhea, and herpes simplex [10, 11].

Only limited data are available on potential connections between *Mg* and HPV [12–17], with studies reporting inconsistent associations between Mg infection and HPV prevalence or persistence and no clear evidence of a direct biological interaction. Microbiological methods are generally used in the diagnosis of *Mg,* but serological assays have also been utilized in seroepidemiological studies [18, 19].

The serological response to *Mg* infection has been tested to define the point-prevalence of *Mg*- seropositivity, but long-term follow-up studies by repeated testing of *Mg* antibody levels are lacking [15, 20]. The two most frequently used *Mg* antigens in serological assays are MgPa N-term and rMgPa, which represent different epitopes of the same major adhesin protein, MgPa, making them suitable targets for detecting antibodies in infected individuals. MgPa protein is the primary virulence factor of *Mg,* playing an essential role in mediating the attachment of the bacteria to host cells, thus facilitating their subsequent invasion [20–23]. In the context of this study, serological testing provides an indirect marker of prior or persistent *Mg* exposure, which may help explore potential associations between past *Mg* infection and HPV serostatus or persistence. As to HPV, antibodies targeting the HPV major capsid protein (L1) are used as an indicator of past exposure and current HPV infections [24]. In our longitudinal study, measuring natural HPV antibody levels allows us to identify prior HPV exposure and monitor infection dynamics over time. However, these natural HPV antibodies remain at substantially lower levels as compared with those achieved by HPV vaccination [25].

The primary objective of this study was to explore the potential interactions between Mycoplasma genitalium (Mg) and human papillomavirus (HPV) using both serology and DNA testing. We assessed the longitudinal outcomes of oral and genital HPV infections among marital couples who were prospectively followed for three years in the Finnish Family HPV (FFHPV) study.

## Material and methods

### Subjects

The Finnish Family HPV Study (FFHPV) is a prospective cohort study jointly conducted at the Department of Gynecology & Obstetrics at Turku University Hospital and the Department of Oral Pathology, University of Turku, Finland. At the study onset, 329 families were enrolled between 1998 and 2002, comprising pregnant women (in 3^rd^ trimester) (*n* = 329), their spouses (*n* = 135), and their newborns (*n* = 331) since the delivery. Not all spouses participated in the study, and the sample included two sets of twins. All study subjects were prospectively followed up for six years by regular clinical visits and multiple sampling for testing of HPV and other infectious agents, as detailed in previous reports [26–29] The FFHPV cohort was originally designed for elucidating the dynamics of HPV infection within regular families but subsequently expanded to analyze other infectious agents as well [26–29]. The Research Ethics Committee of Turku University Hospital has approved this study design and its amendments (#3/1998, 2/2006, and 45/1801/2018), and this study was performed in line with the principles of the Declaration of Helsinki. Informed written consent to participate to this study was obtained from all participants of the study.

### Samples

All study subjects were tested for genital and oral HPV infections at baseline and 2-, 6-, 12-, 24-, 36-, and 72-month visits as previously described, with slightly different sampling strategies for mothers, fathers, and newborns. Scrapings included the use of cytobrush on the uterine cervix (Cytobrush, MedScand, Sweden) and scrapings of oral buccal mucosa of both cheeks and lower vestibular area by using a small brush, ensuring thorough collection of epithelial cells while avoiding touching the tongue as previously described. [26, 28, 30] All samples were immediately frozen at −20 °C and subsequently stored at −70 °C until analyzed.

### Cervical Pap smear cytology, colposcopy, and cervical biopsies

A routine Papanicolaou (Pap) smear was obtained from all women at baseline and at 12-, 24-, and 36-month visits using the conventional 3-sample technique [28]. The smears were evaluated at the Department of Pathology and classified according to the Bethesda System (TBS). Women with abnormal results were referred for colposcopy at the Department of Gynecology and, when indicated, cervical biopsies were obtained and graded by the level of cervical intraepithelial neoplasia as CIN I–III following standard diagnostic practice [28].

### HPV genotyping

The HPV genotyping was done using the Luminex bead-based multiplex papillomavirus genotyping as previously described [26]. Altogether, 24 different HR- (HPV types 16, 18, 26, 31, 33, 35, 39, 45, 51, 52, 53, 56, 58, 59, 66, 68, 73, and 82) and LR- (HPV types 6, 11, 42, 43, 44, and 70) genotypes were detected. These genotypes were selected as they represent the most prevalent and clinically relevant HPV types and include those most commonly associated with cervical and other anogenital and oropharyngeal cancers.

### Serological assay for HPV and *M. genitalium*

Serum blood samples for serological assays were collected at baseline and 12-, 24-, and, 36-month follow-up visits. The serological assays were performed at the German Cancer Research Center (DKFZ), Heidelberg, Germany. The quantitative multiplex serology assay was used for both *HPV* and *M. genitalium* antibodies, as previously described [31, 32]. The method is based on a glutathione S-transferase (GST) capture fluorescent-bead technology.

For HPV serology, the major capsid protein L1 serves as the antigen for serological testing of the LR-HPV types (HPV6, HPV11) and HR-HPV types (HPV16, HPV18, and HPV45). The median fluorescence intensity (MFI) of at least 100 beads per antigen was measured, and sera were scored positive for HPV when the antigen-specific MFI values exceeded the cut-off level of 200 for the L1 antigen of individual HPV types [24].

Serology to *Mg* was analyzed using the primary virulence factor of this bacterium, MgPa, in two protein fragments: MgPa N-Term and rMgPa [21, 22]. The development and validation of the assay have been described in detail earlier [15, 20]. The cut-off for *Mg* was MFI > 1000 for both MgPa N-term and rMgPa, and *Mg* serology was considered positive when the MFI readings of both *Mg* antigens exceeded these cut-offs. The determination of these cut-off values has been thoroughly described by Trabert et al. [15].

### Serological outcomes

Altogether, 281 women and 119 men who had at least two blood samples available were originally included in this study. However, 6 of the fathers and 17 mothers were excluded due to inconsistent *Mg* antibody results that fluctuated between seropositive and seronegative, to minimize potential bias from assay variability and focus on reliably classified cases. The remaining cohort of 264 mothers and 113 fathers was categorized according to their serological outcomes as 1) always negative subgroup (*n* = 225), consisting of individuals whose antibody levels remained below the defined cut-off value at every follow-up visit, and 2) always persistent subgroup (*n* = 35), including the subjects whose seropositivity persisted throughout the entire follow-up time. Positive (seronegative to -positive) seroconversion (*n* = 4) and antibody decay (positive to negative, *n* = 5) groups were recorded but not used in the analysis because of their small number of cases.

*Mg* seropositivity was further stratified into tertiles based on the mean antibody titers at all follow-up visits: i) consistently negative (MFI < 1000), ii) low antibody levels, and iii) high antibody levels (MgPa N-term MFI > 3319, rMgPa MFI > 3647). HPV genotypes were also categorized using the distinction between HR- and LR-HPV genotypes. Persistent oral and genital HPV infections were defined as cases testing HPV positive at two or more consecutive follow-up visits, corresponding to persistence for more than 12 or 24 months. Persistent HPV seropositivity was defined as cases remaining HPV-seropositive at all follow-up visits, with mean fluorescence intensity MFI values consistently exceeding 200.

### Statistical analysis

Statistical analyses were performed using STATA MP17.0 (StataCorp, College Station, TX, USA). For categorical variables, comparisons were made using the χ^2^ test with the likelihood ratio or Fisher’s exact test, depending on sample size. For continuous variables, differences in means were evaluated using nonparametric tests (Mann–Whitney U or Kruskal–Wallis), as the data were not normally distributed. Associations were quantified using regression models. Crude odds ratios (ORs) and 95% confidence intervals (CIs) were obtained by univariate logistic regression, while adjusted ORs were derived from multivariate models. Prevalence ratios (PRs) and their 95% CIs were estimated using multivariate Poisson regression with robust variance to account for common outcomes. All tests were two-sided, and p-values < 0.05 were considered statistically significant.

## Results

**Fig. 1:**
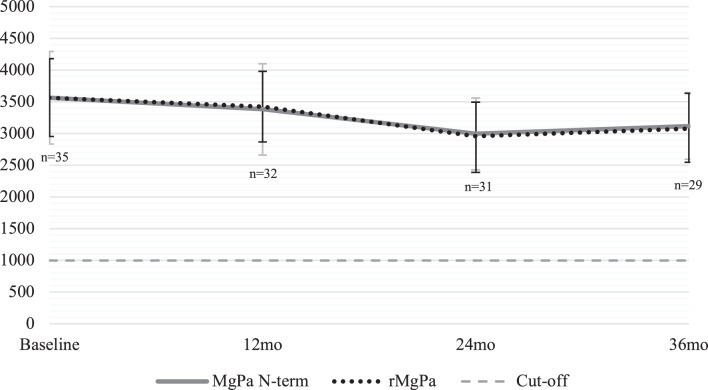
Mean MFI (and the 95% CI) of the IgG-antibody titers to *M. genitalium* antigens MgPa N-term and rMgpa among the seropersistent women during the three-year follow-up using the ANOVA test

Altogether, 13.3% (*n* = 35) of the women were persistently *Mg-*seropositive, whereas 85.2% (*n* = 225) of the women were always *Mg*-seronegative during the three-year follow-up. The mean antibodies to *Mg* antigens MgPa N-term and rMgPa were highly concordant and showed no significant differences in titer levels in any of the visits during the three-year follow-up (Fig. 1). Additionally, both antigens exhibited consistent stability over the follow-up period, as mean MFI levels remained between 2994.0–3563.4 for MgPa N-term and 2958.3–3567.0 for rMgPa.

Persistent oral and genital HPV infections were detected among 25.4% (*n* = 67) and 39.8% (*n* = 105) of the 264 women, respectively. Of the 34 women who exhibited seropersistence to *Mg* and had HPV data available, 11 (32.4%) exhibited persistent oral HR-HPV16 infection (OR 2.02; 95%CI 0.81–5.06), while 10 (29.4%) were always oral HPV negative (Table 1). Among the 224 women who were always *Mg*-seronegative, HR-HPV types 16 and 18 were the most prevalent in the oral mucosa, and 46.0% tested consistently HPV negative. Similar patterns were observed in the genital tract, with HR-HPV types predominating. Persistent *Mg* seropositivity was associated with genital LR-HPV (OR 2.07; 95%CI 0.18–24.0) and HR-HPV (OR 1.08; 95%CI 0.36–3.22), however, these were not statistically significant. Additionally, no statistically significant prevalence ratios were observed between *Mg* seropersistence and the different HPV statuses. Oral and genital HR-HPV infections’ PRs were 1.86 (95%CI 0.83–4.14) and 1.07 (95%CI 0.42–2.84), respectively.

**Table 1:** Prevalence ratios (PR) of *M. genitalium* (*Mg*) seropersistence stratified by oral and genital HPV outcomes using multivariate Poisson regression model with robust variance among the women

HPV infection outcomes	Oral				Genital			
	Mg neg	Mg pos	PR	95%CI	Mg neg	Mg pos	PR	95%CI
	*n* = 224*	*n* = 34*			*n* = 204*	*n* = 30*		
	n (%)	n (%)			n (%)	n (%)		
HPV always neg	103 (46.0)	10 (29.4)	ref	ref	31 (15.2)	5 (16.7)	ref	ref
Fluctuating HPV **	65 (29.0)	13 (38.2)	1.88	0.87–4.08	84 (41.2)	9 (30.0)	0.70	0.25–1.94
Persistent LR-HPV	–	–	–	–	3 (1.47)	1 (3.3)	0.27	0.27–11.87
Persistent HR-HPV ***	56 (25.0)	11 (32.4)	1.86	0.83–4.14	86 (42.2)	15 (50.0)	1.07	0.42–2.84

* Number of *Mg* seropersistent/seronegative participants, who also had at least two HPV samples from the corresponding anatomical site

** Including incident HPV or HPV clearance

*** Those women who had persistent LR- and HR-HPV infection were included in the HR-HPV group

From the fathers, only the oral HPV data was available for these analyses (Supplementary Table 1). Altogether, 104 men (92.0%) were always seronegative to *Mg*, and only 6 men showed persistent seropositivity to *Mg* (5.3%). Additionally, there were only four couples in which both partners were seropersistent to *Mg* during the whole follow-up period. Persistent oral HPV infections were exclusively observed in men exhibiting persistent seronegativity to *Mg*, most of them being HR-HPV infections. Of the 102 men who were consistently seronegativity to *Mg* and had HPV data available, 43 (42.2%) exhibited also HPV negativity, while 30 (29.4%) had persistent single or multiple HR-HPV infection. Those 6 men who were *Mg* seropersistent, were either HPV negative (*n* = 4, 66.7%) or had fluctuating HPV results (*n* = 2, 33.3%). Given the small number of *Mg*-seropositive men, statistical power was limited, restricting further evaluation of *Mg*–HPV associations in males.

In women, we examined the potential associations of the *Mg* antibody tertiles to the incident oral and genital HPV infections as well as HPV clearance during the follow-up (Table 2). Only the women exhibiting the highest levels of MgPa N-term antibodies (MFI > 3319) displayed an increased likelihood of incident oral HPV infections seen during the three-year follow-up, with crude OR 4.14 (95%CI 1.10–15.52) and when adjusted with the number of lifetime sexual partners, adjusted OR 4.33 (95%CI 1.14–16.48), *p* = 0.032. Sexual behavior was considered a potential confounding factor, given that both *HPV* and *Mg* are sexually transmitted. All other associations between the different levels of *Mg* antibodies and HPV incidence or clearance were not statistically significant.

**Table 2:** *M. genitalium (Mg)* antibody tertiles associations with incident HPV and HPV clearance among women*

		Incident HPV infection^h^		HPV clearance^i^	
		Oral *n* = 115	Genital *n* = 203	Oral *n* = 77	Genital *n* = 130
*Mg* antigen	MFI-tertiles	OR (95%Cl)			
MgPa N-term	neg^a^	1.00	1.00	1.00	1.00
	low^b^	1.42 (0.49–4.07)	0.68 (0.18–2.60)	0.82 (0.20–3.29)	0.83 (0.21–3.34)
	high^c^	4.14 (1.10–15.52) **	0.84 (0.17–4.07)	3.81 (0.92–15.85)	1.09 (0.22–5.55)
rMgPa	neg^a^	1.00	1.00	1.00	1.00
	low^d^	2.23 (0.72–6.92)	2.04 (0.26–16.47)	1.53 (0.39–5.92)	3.75 (0.47–30.04)
	high^e^	2.23 (0.72–6.92)	0.42 (0.12–1.45)	1.91 (0.53–-6.88)	0.23 (0.05–1.11)
Both antigens	neg^a^	1.00	1.00	1.00	1.00
	low^f^	1.55 (0.40–5.96)	1.49 (0.18–12.36)	1.43 (0.31–6.62)	2.19 (0.26–18.50)
	high^g^	3.72 (0.73–18.93)	0.56 (0.11–2.90)	4.77 (0.90–25.40)	0.31 (0.04–2.31)

* Using the univariate logistic regression model

** When used multivariate analysis and adjusted with number of lifetime sex partners, OR 4.33 (95%CI 1.14–16.48), p = 0.032

^a^ MFI < 1000 is the cut-off for seronegativity

^b^ 1000 < MFI ≤ 3319 is the range for low MgPa N-term MFI-tertial

^c^ MFI > 3319 is the cut-off for high MgPa N-term MFI-tertial

^d^ 1000 < MFI ≤ 3647 is the range for low rMgPa MFI-tertial

^e^ MFI > 3647 is the cut-off for high rMgPa MFI-tertial

^f^ Both MgPa N-term and rMgPa MFI-tertiles are low

^g^ Both MgPa N-term and rMgPa MFI-tertiles are high

^h^ Participants whose baseline was negative, but HPV DNA was detected later during the follow-up

^i^ Participants who cleared the infection during follow-up and were negative at last visit

Table 3 depicts the associations of the MgPa N-term and rMgPa antibody tertiles to short (12-month) and long-term (24-month) persistence of oral and genital HPV16 infections and the development of incident CIN during the follow-up. There is a tendency for 24-month persistent genital HPV16 infections to be more frequent among women displaying the highest levels of *Mg* antibodies (MgPa N-term MFI > 3319, rMgPa MFI > 3647) as compared to *Mg*-seronegative women, although the statistical significance was not reached. The p-values ranged from 0.65 to 0.76. These findings from Tables 2 and 3 may suggest that stronger or persistent *Mg*-specific immune responses could reflect underlying mucosal immune modulation or epithelial inflammation, potentially facilitating HPV persistence or enhancing susceptibility to infection.

**Table 3:** *M. genitalium (Mg)* antibody tertiles associations with short- and long-term persistent HPV16 infections and incident CIN*

		Over12-mo persistence**		Over 24-mo persistence***		Progression to CIN
		Oral	Genital	Oral	Genital	
*Mg* antigen	MFI-tertiles OR (95%Cl)					
MgPa N-term	neg^a^	1.00	1.00	1.00	1.00	1.00
	low^b^	1.73 (0.55–5.42)	0.87 (0.21–3.72)	2.55 (0.15–44.37)	0.89 (0.05–15.00)	4.13 (0.23–73.29)
	high^c^	3.37 (0.77–14.64)	0.65 (0.10–4.12)	0.51 (0.05–4.87)	1.33 (0.21–8.67)	…
rMgPa	neg^a^	1.00	1.00	1.00	1.00	1.00
	low^d^	2.42 (0.71–8.32)	2.62 (0.30–22.69)	1.27 (0.10–15.50)	0.89 (0.12–6.81)	…
	high^e^	2.02 (0.56–7.29)	0.33 (0.07–1.55)	0.64 (0.06–6.34)	1.78 (0.15–20.86)	1.38 (0.13–15.03)
Both antigens	neg^a^	1.00	1.00	1.00	1.00	1.00
	low^f^	2.02 (0.49–8.41)	2.18 (0.24–19.47)	2.55 (0.15–44.37)	0.89 (0.05–15.00)	…
	high ^g^	3.03 (0.49–18.7)	0.47 (0.06–3.24)	0.64 (0.06–6.34)	1.78 (0.15–20.86)	…

* Using the univariate logistic regression model

** Always negative as the reference

*** < 24-month persistence or clearance as the reference

^a^ MFI < 1000 is the cut-off for seronegativity

^b^ 1000 < MFI ≤ 3319 is the range for low MgPa N-term MFI-tertial

^c^ MFI > 3319 is the cut-off for high MgPa N-term MFI-tertial

^d^ 1000 < MFI ≤ 3647 is the range for low rMgPa MFI-tertial

^e^ MFI > 3647 is the cut-off for high rMgPa MFI-tertial

^f^ Both MgPa N-term and rMgPa MFI-tertiles are low

^g^ Both MgPa N-term and rMgPa MFI-tertiles are high

The analysis of *Mg* antibody tertiles and HPV serology outcomes showed that consistent HPV seronegativity was rare among *Mg*-seropositive women (*n* = 3, 7.1%) compared with those who were always *Mg*-seronegative (*n* = 39, 92.9%). Although no statistically significant differences were observed between *Mg* antibody levels and HPV serology outcomes (Supplementary Table 2), a stronger trend between persistent *Mg* seropositivity and HPV seropositivity across follow-up timepoints was noted (Table 4).

**Table 4:** Overall M. genitalium (Mg)* seropositivity associations with HR- and LR-HPV** seropositivity compared to HPV seronegative participants per follow-up visits among women***

	HPV Seropositivity per follow-up visit	
*Mg* Seropositivity	Any LR-HPV^a^	Any HR-HPV^b^
Visit	OR (95%CI)	
Baseline	**2.73 (1.06–7.04)**	**4.62 (1.93–11.0)**
12mo	1.65 (0.58–4.67)	2.10 (0.81–5.47)
24mo	1.47 (0.51–4.20)	**2.66 (1.01–7.00)**
36mo	0.64 (0.21–1.97)	**2.85 (1.26–6.42)**

* MFI ≥ 1000 as the cut-off for Mg seropositivity for both antibodies MgPa N-term and rMgPa

** Low-risk HPV (LR-HPV) includes serotypes 6 and 11, and high-risk HPV (HR-HPV) includes serotypes 16, 18 and 45 in this analysis; cut-off for seropositivity was MFI > 200

*** Using the univariate logistic regression model

^a^ Low-risk HPV (LR-HPV) includes serotypes 6 and 11 in this analysis

^b^ High-risk HPV (HR-HPV) includes serotypes 16, 18 and 45 in this analysis. Those who were seropositive to LR- and HR-HPV were included in the HR-group

Specifically, *Mg* and HR-HPV seropositivity were significantly associated at baseline, 24 months, and 36 months, with ORs ranging from 2.66 to 4.62 (95%CI 1.01–11.0). At baseline, LR-HPV seropositivity was also significantly associated with *Mg* seropositivity (OR 2.73; 95% CI 1.06–7.04). When only high *Mg* antibody titers were considered, the baseline associations with both LR- and HR-HPV seropositivity became stronger (ORs 8.53 and 4.12; 95%CI 1.04–68.45) (Supplementary Table 2).

## Discussion

This study examined the associations between *Mg* antibody levels and the outcomes of oral and genital HPV infections, assessed both by DNA testing and serological responses, in a longitudinal cohort of marital couples. To our knowledge, this is the first study to explore these interactions using a combined serological and molecular approach. The key finding was that women with persistent *Mg* antibodies had a markedly higher likelihood of being HR-HPV seropositive during the three-year follow-up. *Mg* seropositivity was significantly correlated with HR-HPV seropositivity at baseline (OR 4.62; 95%CI 1.93–11.0), 24 months (OR 2.66; 95%CI 1.01–7.00), and 36 months (OR 2.85; 95%CI 1.26–6.42). Because of the observational nature of this study, the temporal relationship between *Mg* and HPV serological responses cannot be established, and causal inference cannot be made. However, women seropositive for HR-HPV types were more likely to show high and stable Mg antibody titers over time.

Some previous studies have explored the relationship between *Mg* and genital HPV infections, reporting inconsistent results [12, 14, 16, 17, 33–35]. These discrepancies likely stem from differences in study design, population characteristics, regional variation, and small sample sizes, as there is still a lack of large, well-designed cohort studies addressing this topic. Importantly, these previous studies have primarily focused on clinical *Mg* infections rather than analyzing the serological responses and their outcomes. These findings suggest that *Mg*, like other sexually transmitted infections, may exert an indirect influence on susceptibility to other pathogens through cellular stimulation that enhances epithelial sensitivity to infection, such as with HPV. In addition, *Mg* may have a more direct effect by suppressing cell-mediated immune (CMI) responses, thereby facilitating the persistence of intracellular pathogens or promoting microbial colonization [36–38].

Unlike direct pathogen detection methods, which are limited to identifying active infections or recent exposures, serological testing reflects long-term immune responses, allowing us to assess historical exposure that may shape the host’s susceptibility to different pathogens [39]. Studies on *Mg* serology are surprisingly rare. A recent study reported that *Mg* seropositivity was more prevalent among women within infertile couples (5.4%) as compared to fertile couples (1.6%), displaying an OR of 3.45 (95%CI 1.10–10.75) [40]. Additionally, few studies have investigated the relationship between Mg antibodies and epithelial ovarian tumors, failing to demonstrate any link between ovarian cancer and *Mg* antibodies [19, 41, 42]. To our knowledge, no previous studies have examined the association between Mg antibodies and HPV infections, HPV-related carcinogenesis, or the co-existence of Mg and HPV serum antibodies.

HPV-induced oral infections are well documented and relatively common [43], whereas *Mg* carriage in the oropharynx appears to be rare, and transmission through oral sex is considered uncommon [3, 44, 45]. This rarity complicates efforts to explore associations between oral HPV infections and *Mg* serology. In our study, oral HPV infections among women were predominantly caused by HPV16, the important high-risk type known to induce dysplastic and potentially malignant lesions in the head and neck region [46]. The present data disclosed that women with higher levels of *Mg* antibodies might be at an increased risk of contracting incident oral HPV infections (Table 2). This association was significant only for the high MgPa N-term antibody levels (OR 4.14; 95%CI 1.10–15.52), but was also significant when adjusted with the number of lifetime sexual partners (OR 4.33; 95%CI 1.14–16.48). When the two antibodies were combined, the OR remained still high, but due to the wide 95%CI, this association did not reach statistical significance.

Women with Mg antibodies showed a higher prevalence of both LR- and HR-HPV antibodies at baseline and, for HR-HPV, across most follow-up visits. This correlation likely reflects their shared mode of transmission. Among women in the highest tertile of MgPa N-term antibodies, 64.7% (*n* = 11) reported more than five lifetime sexual partners, a trend also observed among HPV-seropositive women. Due to the limited sample size, we could not confirm the impact of *Mg* antibodies on the persistence of HPV antibodies. The inherent instability of natural HPV antibodies may further obscure potential long-term associations between *Mg* and HPV serology.

In the FFHPV cohort, the number of male participants is substantially lower than that of women (26–29), which also affected the stratified analyses in the present study. Among the male population in general, non-gonococcal urethritis (NGU) stands as the predominant clinical presentation of *Mg* infection. However, the precise prevalence of NGU among the *Mg*-infected men remains undetermined, albeit estimated to affect only a minority of afflicted individuals. The prevalence of *Mg* among women and men is concordant, leveling off at around 1% [3]. The role of serological testing of *Mg* remains relatively unexplored, and the studies elucidating the potential correlations between *Mg* seropositivity and HPV infection among males are practically non-existent. Nevertheless, given the shared transmission routes and risk factors [3], it is possible that similar Mg–HPV associations could exist in men. However, the small male sample size restricts statistical power, limiting the ability to detect potential associations and necessitating cautious interpretation of these findings.

Our study has many strengths, alongside with limitations. We believe that the cohort size of the women was adequate, permitting a comprehensive exploration of the relationships between *Mg* antibodies and HPV serology as well as HPV oral and genital infections, even when stratified by the *Mg*-antibody levels. An additional strength of our novel study is the longitudinal study design, with a prospective follow-up of three years by multiple serial samplings. When it comes to the limitations of this study, the major limitations of this study were the small number of male participants, which limited analyses in men. Additionally, the lack of *Mg* infection data confirmed by nucleic acid amplification tests (NAATs) during follow-up prevented a more precise assessment of the relationship between infection and serological responses, as NAATs are needed to confirm current infection rather than just past exposure. Minor limitations involve the limited ability to examine mechanisms of seroconversion or antibody decay and, for certain analyses among women, a smaller sample size than ideal.

## Conclusions

In summary, this study demonstrates a close serological association between HPV and *Mg* antibodies. While consistent associations between *Mg* antibodies and oral or genital HPV infections were not observed, the potential link warrants further investigation. Future research should involve larger, longitudinal cohorts including both genders, integrate molecular confirmation of *Mg* infections (e.g., NAATs), and explore the mechanistic pathways underlying *Mg*–HPV interactions. Such studies will be essential for clarifying the role of *Mg* in HPV acquisition and persistence, and for guiding targeted prevention and intervention strategies.

## Electronic supplementary material

Below is the link to the electronic supplementary material.

Supplementary Material 1

## Data Availability

The datasets used and/or analyzed during the current study are available from the corresponding author upon reasonable request.

## Abbreviations

STIs: Sexually transmitted infections
Ct: Chlamydia trachomatis
Mg: Mycoplasma genitalium
PID: Pelvic inflammatory disease
NGU: Non-gonococcal urethritis
HR-HPV: High-risk HPV
LR-HPV: Low-risk HPV
pGp3: PGP3-D-CT
rMgPa: Recombinant MgPa
IgG: Immunoglobulin G
CIN: Cervical intraepithelial neoplasia
GST: Glutathione S-transferase
The FFHPV: The Finnish Family HVP Study
MFI: Median fluorescence intensity
OR: Odds ratio
CI: Confidence interval
CMI-response: Cell-mediated immune response
