# Supplementary material for: Interactions of Mycoplasma genitalium (Mg) and human papillomavirus (HPV) infections among couples
Source: BMC Infect Dis. 2025 Dec 23;26:163. doi: 10.1186/s12879-025-12334-y (PMC12837238; doi:10.1186/s12879-025-12334-y)
Supplement: Supplementary file 1 — Supplementary Material 1 [file 12879_2025_12334_MOESM1_ESM.docx]

**Supplementary Material**

| **Supplementary Table 1.** *M. genitalium* (*Mg*) seropersistence stratified by oral HPV outcomes among the men. | | |
| --- | --- | --- |
|  | Mg neg | Mg pos |
| Oral HPV infections | n=102* | n=6* |
|  | n (%) | n (%) |
| HPV always neg | 43 (42.2) | 4 (66.7) |
| Fluctuating HPV ** | 27 (26.5) | 2 (33.3) |
| Persistent LR-HPV | 2 (2.0) | … |
| Persistent HR-HPV *** | 30 (29.4) | … |
| * Number of Mg seropersistent/seronegative participants, who also had at least two HPV samples.  ** Including incident HPV or HPV clearance.  *** Those who had persistent LR- and HR-HPV infection were included in the HR-HPV group. | | |

| **Supplementary Table 2.** *M. genitalium (Mg)* antibody tertiles association with serological HPV outcomes among women*. | | | | | |
| --- | --- | --- | --- | --- | --- |
|  |  | Baseline HPV Seropositivity | | HPV serology outcomes during FU | |
|  |  | LR-HPV ^i^ | HR-HPV ^j^ | Always seronegative ^k^ | Persistent antibody levels ^l^ |
| *Mg* antigen | MFI-tertiles | OR (95% Cl) | | | |
| MgPa N-term | neg ^a^ | 1.00 | 1.00 | 1.00 | 1.00 |
|  | low ^b^ | 1.18 (0.45–3.11) | 2.21 (0.84–5.81) | 0.28 (0.04–2.17) | … |
|  | high ^c^ | 2.27 (0.78–6.67) | 1.99 (0.74–5.35) | 0.64 (0.14–2.89) | 0.85 (0.05–14.33) |
| rMgPa | neg ^a^ | 1.00 | 1.00 | 1.00 | 1.00 |
|  | low ^d^ | 0.95 (0.36–2.48) | 1.12 (0.42–3.01) | 0.60 (0.13–2.70) | 1.69 (0.14–19.94) |
|  | high ^e^ | 3.08 (0.97–9.74) | **4.24 (1.44–12.46)** | 0.30 (0.04–2.31) | … |
| Both antigens | neg ^a^ | 1.00 | 1.00 | 1.00 | 1.00 |
|  | low ^f^ | 1.14 (0.34–3.83) | 1.47 (0.44–4.97) | 0.48 (0.06–3.83) | … |
|  | high ^g^ | **8.53 (1.06–68.45)** | **4.12 (1.04–16.37)** | 0.53 (0.07–4.30) | … |
| ** Using the univariate logistic regression model.*  ^a^ *MFI < 1000 is the cut-off for seronegativity.*  ^b^ *1000 < MFI ≤ 3319 is the range for low MgPa N-term MFI-tertial.*  ^c^ *MFI >3319 is the cut-off for high MgPa N-term MFI-tertial.*  ^d^ *1000 < MFI ≤ 3647 is the range for low rMgPa MFI-tertial.*  ^e^ *MFI >3647 is the cut-off for high rMgPa MFI-tertial.*  ^f^ *Both* *MgPa N-term and rMgPa MFI-tertiles are low.*  ^g^ *Both* *MgPa N-term and rMgPa MFI-tertiles are high.*  ^i^ *Low-risk HPV (LR-HPV) includes serotypes 6 and 11 in this analysis.*  ^j^ *High-risk HPV (HR-HPV) includes serotypes 16, 18 and 45 in this analysis.*  ^k^ *Participants who were always seronegative to all five HPV-types (HPV6, -11, -16, -18 and 45).*  ^l^ *Participants who always had high HPV antibody levels, MFI>200.* | | | | | |
|  |  |  |  |  |  |
